# Two‐conformer equilibrium of maltose‐binding protein in the absence of ligand from residual dipolar coupling analysis

**DOI:** 10.1002/pro.70425

**Published:** 2025-12-23

**Authors:** Yang Shen, Ad Bax

**Affiliations:** ^1^ Laboratory of Chemical Physics National Institute of Diabetes and Digestive and Kidney Diseases, National Institutes of Health Bethesda Maryland USA

**Keywords:** allostery, conformational equilibrium, excited state, protein NMR, RDC

## Abstract

Prior analyses found good agreement between numerous residual dipolar couplings (RDCs) measured in the apo‐state of maltose binding protein (MBP) and its X‐ray crystal structure. However, paramagnetic relaxation enhancement (PRE) measurements on the same system reported on the presence of a small population of partially closed states in the absence of ligand, with somewhat different relative orientations of its N‐ and C‐terminal domains. We present a protocol for RDC fitting to such a dynamic system that yielded quantitative validation of the PRE results. Our analysis is based on a multi‐conformer singular value decomposition (SVD) RDC fitting procedure that provides a straightforward method for quantifying conformational equilibria, provided that high‐quality RDCs and accurate coordinates for invariant domains of the protein are available, such as may apply for allosterically regulated systems. For MBP, the analysis reveals interdomain dynamics that can be fit to a two‐state equilibrium, with the major state very close to the apo‐state X‐ray structure and a minor conformer near the center of an ensemble of partially closed structures, previously derived by PRE. The holo‐state of MBP was found to agree, to within RDC measurement precision, with a single relative orientation of its two domains. The multi‐tensor multi‐conformer software is available to all users as an interactive web application at https://spin.niddk.nih.gov/bax-apps/nmrserver/dc/svdm.html.

## 1 INTRODUCTION

Residual dipolar couplings (RDCs), measured by solution NMR spectroscopy, contain precise information on bond vector orientations in proteins and nucleic acids (Chiliveri et al. 2022; Prestegard et al. 2004; Tjandra and Bax 1997; Tolman et al. 1995). Although measurement of RDCs often remains limited to the largest internuclear interactions, such as the one‐bond ^1^D_CH_ and ^1^D_NH_ couplings, intrinsically much smaller RDCs such as ^1^D_C′N_, ^1^D_C′Cα_, ^1^D_CαCβ_, ^2^D_C′HN_, and ^3^D_C′Hα_ can also be measured at high precision in proteins (Evenäs et al. 2001b; Skrynnikov et al. 2000; Wang et al. 1998; Yang et al. 1999). In fact, ^1^D_C′N_ and ^1^D_C′Cα_ can yield better agreement with X‐ray structures than the more widely used ^1^D_NH_ RDCs that suffer from small uncertainties in the N‐H vector orientation when the ^1^H coordinates are added to an X‐ray structure under the assumption of standard, in‐peptide‐plane geometry (Ottiger and Bax 1998; Yang et al. 1999).

A large set of ^1^D_NH_, ^1^D_C′N_, ^1^D_C′Cα_, ^1^D_CαCβ_, ^2^D_C′HN_, and ^3^D_C′Hα_ RDCs was previously measured for maltose binding protein (MBP) (Evenäs et al. 2001a; Yang et al. 1999) and used to assess the relative orientation of its two globular domains in the apo‐ and holo‐states of the protein (Evenäs et al. 2001a; Evenäs et al. 2001b; Skrynnikov et al. 2000). While solution RDCs pointed to small differences in relative domain orientation for β‐cyclodextrin‐ligated MBP relative to its X‐ray structure (Evenäs et al. 2001b; Skrynnikov et al. 2000), close agreement between RDCs and the X‐ray structures of the MBP apo‐state was observed, as well as for MBP bound to its natural ligand, maltotriose (Evenäs et al. 2001b).

Remarkably, a subsequent paramagnetic relaxation enhancement (PRE) study of apo‐MBP found a small population of a partially closed apo‐state even in the absence of ligands, pointing to a dynamic equilibrium between open and partially closed apo‐conformations of MBP (Tang et al. 2007). PREs are very sensitive to short distances, and even while long‐range interdomain contacts were highly transient, they were unambiguously observed in the apo‐state, whereas the low population of these transient contacts did not detectably impact the time‐averaged relative domain orientation as observed by RDCs.

Here, we introduce a multi‐tensor, singular value decomposition (SVD)–based protocol for fitting of apo‐MBP RDCs that reveals the presence of the lowly populated, partially closed apo‐conformation of MBP without recourse to PRE, finding a ca. 16% population of a very similar, partially closed conformation. The protocol requires a step that minimizes the impact of structural noise (Zweckstetter and Bax 2002) in the X‐ray data and identification of invariant domains whose coordinates and relative bond vector orientations are highly conserved in multiple X‐ray structures of the protein. Success of the protocol also hinges on the domain motions being of sufficient amplitude and populations of minor conformers being sufficiently large. For such cases, the method may offer a sensitive complement to the use of PRE or chemical shift perturbation for probing multi‐state equilibria in cases where high‐quality atomic coordinates for multiple states are available. Our approach is distinctly different from prior ensemble refinement protocols that aimed to utilize RDCs for characterizing chain motions at the local level (Lange et al. 2008; Lindorff‐Larsen et al. 2005).

## 2 RESULTS

### 2.1 Identification of invariant MBP domains

Protein domains are structurally well‐ordered units whose movement relative to other domain(s) of the same protein is typically key to the protein's function (Wang et al. 2021). The first step in identifying domain reorientations in a protein from RDCs is to define the residues that make up the domains. This procedure requires more stringent residue‐selection criteria than commonly used in structure‐based domain identification methods because the analysis of domain motions from RDCs hinges on the premise that relative orientations of bond vectors in any given domain remain invariant.

For domain identification, we searched for the largest group of residues whose backbone atom coordinates in our set of MBP X‐ray structures (Table 1) could be superimposed to within a given threshold (here selected at 0.5 Å) in differentially ligated states of the protein (see section 4). After removing these residues from the input files, the search was repeated to find the second largest domain.

**TABLE 1:** Agreement between apo‐state RDCs and MBP X‐ray structures.

Ligand	PDB	Resolution (Å)	Closure/twist/bend angle (°)^a^	*Q* (NTD)^b^	*Q* (CTD)^b^	*Q* (NTD)^c^	*Q* (CTD)^c^	*Q* (all)^d^
Apo	1OMP	1.8	0.0/0.0/0.0	0.239	0.221	0.176	0.180	0.180
Apo	1JW4	2.0	−0.6/0.1/−0.6	0.289	0.218	0.205	0.186	0.200
Maltose	*1ANF ^e^	1.67	36.2/−3.7/0.0	0.291	0.205	0.214	0.177	0.638
Maltose	1JW5	2.0	−1.2/0.6/−1.0	0.241	0.231	0.201	0.176	0.192
Maltotriose	*3MBP ^e^	1.7	34.9/−3.8/−0.4	0.262	0.234	0.212	0.184	0.604
Maltotriotol	1FQB	1.9	2.0/−0.4/−2.7	0.219	0.234	0.184	0.180	0.183
Maltotriotol	*1FQC ^e^	2.3	33.4/−2.6/−0.1	0.350	0.276	0.267	0.246	0.598
Maltetrose	*4MBP ^e^	1.7	34.9/−4.5/−0.8	0.260	0.244	0.195	0.189	0.613
Maltotetraotol	* 1FQD ^e^	2.3	35.1/−3.4/0.1	0.461	0.302	0.396	0.259	0.668
Maltotetraotol	1EZ9_A	1.9	5.2/0.9/−1.3	0.215	0.267	0.188	0.175	0.194
Maltotetraotol	1EZ9_B	1.9	0.2/0.8/−0.3	0.215	0.265	0.186	0.185	0.188
β‐cyclodextrin	1DMB	1.8	2.8/0.2/−0.3	0.281	0.300	0.220	0.232	0.232

^a^ Closure, twist, and bend angles of the C‐terminal domain, measured from the orientation of the C‐terminal domain relative to that of the X‐ray structure 1OMP after superimposing their N‐terminal domains (see legend to Figure 1).

^b^ *Q* factor obtained from fitting apo‐RDCs of all residues in the N‐terminal domain (“NTD”; residues 5–105 and 266–313) or C‐terminal domain (“CTD”; residues 115–251 and 334–370) to the raw PDB coordinates.

^c^ *Q* factor for fits of apo‐RDCs to the raw PDB coordinates for residues in NTD and CTD that are invariant between 1OMP and 3MBP (see section 4 for details of the residue selection).

^d^ *Q* factors for fits of apo‐RDCs (invariant residues only) to the PDB coordinates of the full X‐ray structures.

^e^ X‐ray structures with a closed conformation are labeled with an asterisk.

The domains selected in this manner were subjected to a second round of pruning because even when coordinates of an individual domain in two structures match to within 0.5 Å, relative internuclear vector orientations in such a domain can still differ substantially. In this second step, we therefore removed any peptide planes for which the vector orientations with available RDCs differed by more than a root‐mean‐square (RMS) threshold value (selected at 5°) from their averaged orientations. This threshold was applied after the backbone atomic coordinates of all domains, taken from the different X‐ray structures, were best‐fit superimposed. During this process, it became clear that the 2.3 Å resolution X‐ray structures, 1FQC and 1FQD, yielded a relatively large number of outliers in their vector orientations, which can be attributed to the lower resolution of these structures. Therefore, these two structures were excluded from all further analyses.

**FIGURE 1:**
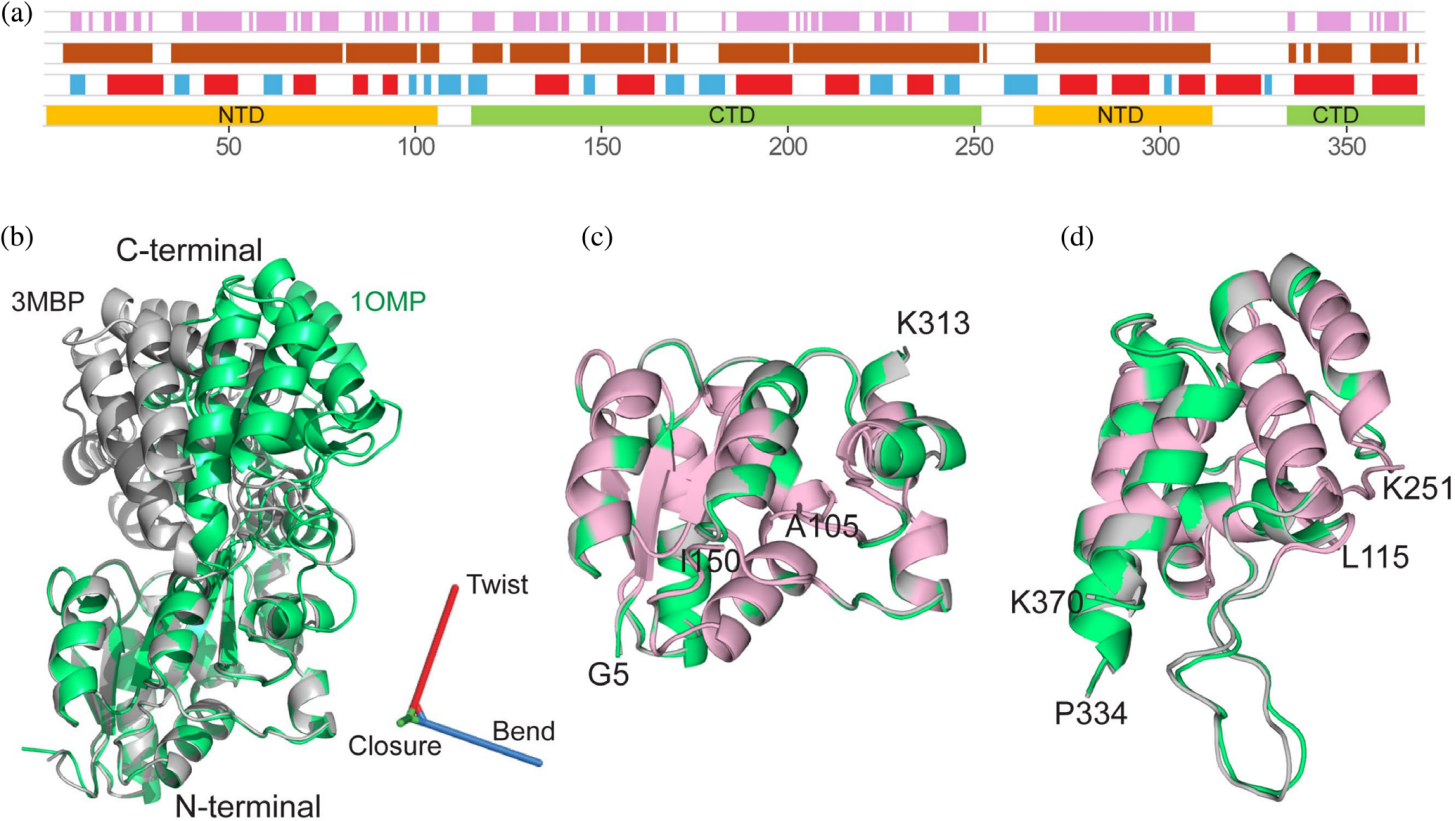
Domain identification and relative orientation in maltose binding protein. (a) Bottom, definition of the N‐terminal (NTD, orange) and C‐terminal (CTD, green) domain of MBP (Tang et al. 2007), with secondary structure marked above it (α‐helices: red; β‐strands: cyan). Residues for which the backbone atoms superimpose to better than 0.5 Å within their respective domains among nine X‐ray structures are shown in brown. Residues within these well‐defined NTD and CTD regions for which N‐H, N‐C′, and C′‐C^α^ bond orientations differ by less than 5° are shown in pink. (b) Ribbon diagrams of apo‐MBP (1OMP, green) and holo‐MBP (3MBP, gray; ligand not shown), with N‐terminal domains of apo and holo MBP superimposed. The orientation of the C‐terminal domain of holo‐MBP is defined relative to the 1OMP reference structure via the sequence of three orthogonal rotations: closure, twist, and bend (Evenäs et al. 2001b). The twist axis (red) is chosen parallel to a line connecting the centers of mass of the 1OMP N‐ and C‐domains; the orthogonal closure axis (green) rotates the C‐domain of 3MBP to that of 1OMP; the bending axis (blue) is formed by the cross‐product of the twist and closure axes. (c, d) Ribbon diagrams of best‐fit superimposed (c) N‐terminal and (d) C‐terminal domains of apo‐MBP (1OMP, green) and holo‐MBP (3MBP, gray), with “invariant” residues (pink bars in panel (a)) shown in pink.

**FIGURE 2:**
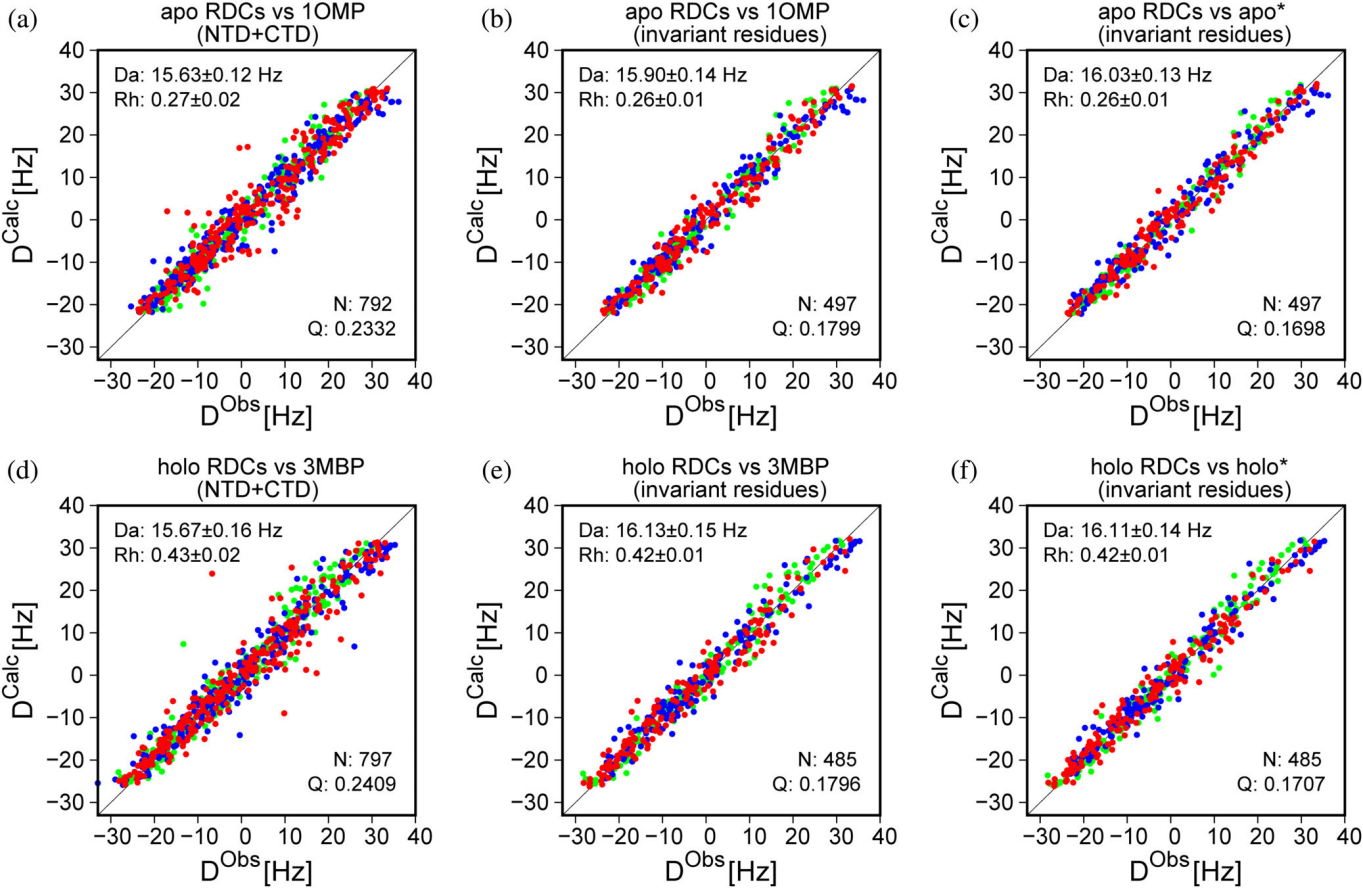
Agreement between RDCs and MBP X‐ray coordinates. Single‐tensor SVD fits were performed for (a–c) Apo RDCs (from Yang et al. 1999) versus apo‐X‐ray structure (1OMP; Sharff et al. 1992), and (d–f) Holo RDCs (from Evenäs et al. 2001b) versus holo X‐ray structure (3MBP; Quiocho et al. 1997). ^1^ *D*_NH_ are shown in red, ^1^ *D*_NC′_ in green, and ^1^ *D*_C′Cα_ in blue, with ^1^ *D*_NC′_ and ^1^ *D*_C′Cα_ upscaled by factors of 5 and 8, respectively, to account for their lower intrinsic dipolar interaction strength. (a, d) Fits performed for all RDCs available in N‐terminal and C‐terminal domains of MBP to (a) 1OMP and (d) 3MBP. (b, e) Analogous fits for the selected invariant residues (see Figure 1a). (c, f) Fits for RDCs of the selected invariant residues after the corresponding vector orientations were replaced by their averaged orientation within the N‐ and C‐domains of 1OMP and 3MBP (see section 4).

Comparison of various apo‐ and holo‐states of MBP identified two invariant domains, with one domain mostly containing residues from the N‐terminal half of its sequence and the second domain predominantly consisting of residues closer in sequence to the C‐terminus (Figure 1). Below, these are referred to as the N‐ and C‐terminal domains. In a third step, the vector orientations used for RDC fits were replaced by orientations of their averaged orientations within the best‐fit superimposed domains. After this replacement, apo‐state RDCs fit better to these averaged orientations (Figure 2a–c) than to any individual structure.

Analogously, RDCs measured in the holo‐state also fit better to the holo‐state X‐ray structure after bond vector orientations within each domain were replaced by their averaged intra‐domain orientations (Figure 2d–f). Similar improvements were observed when averaging vector orientations before fitting RDCs of individual domains rather than the full protein (Figure S1, Supporting Information). Averaging of the vector orientations therefore reduced the structural noise of the MBP X‐ray structures, as judged by improved fits to the RDCs. Therefore, domains with the averaged invariant vector orientations were best‐fit superimposed on the apo‐state (1OMP), to yield the apo* structure. Similarly, superimposing these same domains on the maltotriose‐ligated state (3MBP) was used to generate the holo* structure.

### 2.2 Single‐tensor SVD fit of MBP RDCs

Fitting of observed RDCs to atomic coordinates involves the determination of the five independent values of the symmetric and traceless 3 × 3 Saupe matrix, ***S***, from a strongly overdetermined set of linear equations (Losonczi et al. 1999),

(1) $$D_{\mathrm{pq}}=C_{\mathrm{pq}}\sum_{i,j=\left\{x,y,z\right\}}S_{\mathrm{ij}}\cos\alpha_{i}\cos\alpha_{j},$$

where cos*α*_*i*_ is the direction cosine of the vector *r*_*pq*_, connecting nuclei *p* and *q*, relative to axis *i* of the molecular coordinate frame. *D*_*pq*_ is the dipolar coupling between *p* and *q*, and *C*_*pq*_ denotes the dipolar interaction constant. One‐bond ^13^C‐^13^C dipolar couplings are intrinsically *ca* five times smaller than ^1^ *D*_NH_ couplings, and ^1^ *D*_C′N_ couplings about eight‐fold, while their respective fits to coordinates yielded comparable Pearson correlation coefficients or *Q*‐factors (Yang et al. 1999). Therefore, when carrying out the simultaneous SVD fits of these different types of RDCs to the X‐ray coordinates, experimental *D*_C′N_ and corresponding *C*_C′N_ values were upscaled eight‐fold, and *D*_C′Cα_ together with *C*_C′Cα_ five‐fold, with unscaled values of ^1^ *D*_NH_ and *C*_NH_.

When using conventional single‐alignment‐tensor fitting of all apo‐RDCs to the apo‐state X‐ray coordinates of MBP (1OMP), a good correlation is obtained between the experimentally measured RDCs and the fitted values, with a Pearson's correlation coefficient *R*_*P*_ = 0.975 and *Q* = 0.233 (Figure 2a; see section 4). Comparably good agreement is seen when fitting the RDCs measured for MBP with bound maltotriose (holo‐RDCs) to those of the holo‐state X‐ray structure 3MBP (Figure 2d). These results are consistent with the original conclusion that in solution the apo‐ and holo‐states of MBP closely resemble their respective X‐ray crystal structures (Evenäs et al. 2001b).

Restricting the SVD fits to domain‐invariant residues, identified in the above‐described manner, improves agreement of the RDCs when carrying out the regular single‐tensor SVD fit, both for the apo‐state (Figures 2b and S1e,f) and holo‐state (Figures 2e and S1g,h). A further improvement to the fit is obtained when fitting the apo‐state RDCs to the apo* structure (Figure 2c) and the holo‐state RDCs to the holo* structure (Figure 2f). Notably, however, when fitting the individual domains of apo*, better fits are obtained for its individual domains (Figure S1i,j) than for the full structure (Figure 2c), suggesting small differences in domain orientation in solution relative to the 1OMP X‐ray structure, or the presence of interdomain dynamics.

### 2.3 RDC fit to single, static structures

A single‐tensor SVD fit of the apo‐RDCs to apo* yielded *Q* values of 0.1696, 0.1653, and 0.1698 for the N‐domain, the C‐domain, and the full apo* (Figures S1i,j and 2c), respectively, with very small uncertainties of ±0.52° and ±0.51° for the orientations of the domain alignment frames. We note that the improvement in fits to the individual domains over fitting the full structure remains when using jackknifed *Q*_*jk*_ values (Shen et al. 2023), that is, when evaluating RDCs not used in the fit, and therefore is not dominated by the smaller number of RDCs relative to the five adjustable parameters of the SVD fits (see section 4.1). The inverse cosine of the normalized scalar product P(***S***_Nterm_, ***S***_Cterm_) (Sass et al. 1999) corresponds to a 2.7° difference in the 5D orientation of the C‐terminal relative to the N‐terminal domain. This difference is dominated by small Cartesian space rotations, *R*_*x*_ = 0.1°, *R*_*y*_ = 2.5°, *R*_*z*_ = 1.9°, that make the alignment frame of the C‐terminal domains coincident with that of the N‐terminal domain. These rotations correspond to a small tilt of the C‐domain roughly towards its orientation in the 3MBP holo‐state. The rotations about *x*, *y*, and *z* are equivalent to a single rotation about an axis defined by spherical coordinates *ϕ*_*r*_ and *ψ*_*r*_ which can be derived using standard methods (see https://en.wikipedia.org/wiki/Rotation_matrix#Axis_and_angle). A 2.9° rotation of the C‐domain about this axis resulted in an apo** structure where the alignment tensors for the N‐ and C‐terminal domains were co‐aligned. An RDC fit of this structure yielded *Q* = 0.1691.

In principle, it is conceivable that apo**, which co‐aligns the alignment tensors of N‐ and C‐terminal domains, is not the best single static structure for fitting the RDCs. However, when carrying out a systematic grid search over *x*‐, *y*‐, and *z*‐axis rotations of the C‐domain covering a range of ±5°, the lowest *Q* factor is obtained for zero rotation (Figure S2), confirming that apo** is an optimal reference structure.

When the same analysis was used to fit the holo‐RDCs to holo*, good *Q* values (0.1915 and 0.1470; Figure S1k,l) were again obtained for the individual domains, with small uncertainties in their alignment tensor orientations (±0.76° and ±0.50°), and an intermediate *Q* = 0.1707 for the full holo* (Figure 2f). The inverse cosine of their normalized scalar product, P(***S***_Nterm_, ***S***_Cterm_), points to a difference in their 5D orientations of 1.29°, which approximately corresponds to the sum of their experimental uncertainties. This result indicates that the coordinates of 3MBP match the solution state RDCs to within measurement precision and that a two‐state equilibrium is not needed to fit the holo‐state RDCs.

### 2.4 Two‐tensor SVD fitting of RDCs

A two‐tensor SVD fit of observed RDCs is needed when a protein switches between two conformational states on a time scale that is slower than the rotational correlation time, that is, the time needed to sense alignment, but faster than the chemical shift differences between the two states, that is, when a single set of resonances is observed. In that case, measured RDCs correspond to the population‐weighted average over the two states sampled by the protein,

(2) $$D_{\mathrm{pq}}=C_{\mathrm{pq}}\sum_{h=1,2\;}\sum_{i,j=\left\{x,y,z\right\}}S_{\mathrm{ij},h}^{\prime }\cos\alpha_{i,h}\cos\alpha_{j,h},$$

where the index *h* refers to conformer *h* (*h* = 1,2), and cos*α*_*i*,*h*_ is the direction cosine of *r*_*pq*_ in conformer *h* relative to its coordinate frame, $S_{\mathrm{ij},h}^{\prime }$ is the population‐weighted Saupe matrix for conformer *h*. This two‐tensor fit therefore includes 10 adjustable parameters, $S_{\mathrm{ij},h}^{\prime }$, rather than the five adjustable parameters in a standard SVD fit.

A weighted two‐tensor SVD fit of *N* couplings is then performed to solve the following set of linear equations:

(3) $$\begin{array}{l} \sum_{h=1,2}{\left(\begin{array}{cc} \begin{array}{ccc} A_{\mathrm{yy}}^{\left(1\right)} & A_{\mathrm{zz}}^{\left(1\right)} & A_{\mathrm{xy}}^{\left(1\right)}\\ A_{\mathrm{yy}}^{\left(2\right)} & A_{\mathrm{zz}}^{\left(2\right)} & A_{\mathrm{xy}}^{\left(2\right)} \end{array} & \begin{array}{cc} A_{\mathrm{xz}}^{\left(1\right)} & A_{\mathrm{yz}}^{\left(1\right)}\\ A_{\mathrm{xz}}^{\left(2\right)} & A_{\mathrm{yz}}^{\left(2\right)} \end{array}\\ \begin{array}{ccc} . & . & .\\ . & . & .\\ A_{\mathrm{yy}}^{\left(N\right)} & A_{\mathrm{zz}}^{\left(N\right)} & A_{\mathrm{xy}}^{\left(N\right)} \end{array} & \begin{array}{cc} . & .\\ . & .\\ A_{\mathrm{xz}}^{\left(N\right)} & A_{\mathrm{yz}}^{\left(N\right)} \end{array} \end{array}\right)}_{h}{\left(\begin{array}{c} S_{\mathrm{yy}}^{\prime }\\ S_{\mathrm{zz}}^{\prime }\\ S_{\mathrm{xy}}^{\prime }\\ S_{\mathrm{xz}}^{\prime }\\ S_{\mathrm{yz}}^{\prime } \end{array}\right)}_{h}\\ \;=\left(\begin{array}{c} D^{\left(1\right)}/\varepsilon^{\left(1\right)}\\ D^{\left(2\right)}/\varepsilon^{\left(2\right)}\\ .\\ .\\ D^{\left(N\right)}/\varepsilon^{\left(N\right)} \end{array}\right), \end{array}$$

where $S_{\mathrm{ab},h}^{\prime }$ (*a*,*b* = *x*,*y*,*z*) are the Saupe matrix elements of conformer *h*, and $A_{\mathrm{ab},h}^{\left(i\right)}$ are direction cosine terms in the molecular frame of conformer *h* for the *i*th (*i* = 1 to *N*) dipolar interaction, *D* ^(*i*)^, scaled by its uncertainty ε^(*i*)^, that is,

(4) $$A_{\mathrm{ab}}^{\left(i\right)}=\begin{array}{l} C_{\mathrm{pq}}\{{∆}_{\mathrm{ab}}\left(\cos^{2}\alpha_{a}^{\left(i\right)}-\cos^{2}\alpha_{x}^{\left(i\right)}\right)\\ +\;2\left(1-{∆}_{\mathrm{ab}}\right)\cos\alpha_{a}^{\left(i\right)}\cos\alpha_{x}^{\left(i\right)}\}/\varepsilon^{\left(i\right)}, \end{array}$$

where $\alpha_{a}^{\left(i\right)}$ (*a* = *x*,*y*,*z*) is the angle between the internuclear vector and the *a* axis of the molecular frame and Δ_*ab*_ is the delta function (Burnell and de Lange 1980; Liu and Prestegard 2010). In practice, it is helpful to choose the coordinate frame for the second conformer (*h* = 2) such that one of the protein domains coincides with that of conformer 1. In principle, the multi‐tensor analysis can readily be extended to any number of unique conformers, but the RDC data will rarely suffice for unambiguous characterization of more than two states. We also note that the population of the two states cannot be determined uniquely from the data because the fractional population of each state is already reflected in the value of $S_{\mathrm{ij},h}^{\prime }$ of Equation (2) (Tang et al. 2007). Although methods have been developed that predict the alignment tensor for proteins of known shape, the accuracy of the predicted alignment strength remains rather low (Zweckstetter 2008). Hence, without accurate knowledge of the Saupe matrix for a 100% populated state *h*, only an estimate for the population can be obtained under the assumption that the predicted alignment is correct or that the generalized magnitude of the alignments in each of the two states is roughly the same when 100% populated. The latter applies for apo‐state and various ligated states of MBP (Evenäs et al. 2001b).

To validate the significance of a multi‐tensor fit, we derived the jackknifed quality factor, *Q*_*jk*_ (see section 4). When fitting a large number (*N* >> 5) of RDCs, *Q*_*jk*_ approaches the standard *Q* value obtained when including all RDCs in the SVD fit (Shen et al. 2023).

The two‐tensor fit of Equation (2) involves 10 adjustable parameters, compared to five such parameters in a standard SVD fit of RDCs to coordinates. Jackknifing, where one observed RDC is cyclically omitted from the *N* SVD fits, therefore requires a minimum of 11 RDCs. Considering that the number of independent RDCs available for MBP, even after the above stringent filtering procedure, remains far higher (248 for the N‐domain; 249 for its C‐domain), these data are well‐suited for jackknifed cross‐validation of the two‐tensor fits.

### 2.5 Reliability of two‐state fitting

Before using the experimental MBP RDCs to analyze the presence of a two‐state ensemble, we carried out simulations to evaluate the sensitivity of such an analysis to errors in the experimental RDCs and uncertainties in the atomic coordinates of the reference structure. Simulations were carried out for the case where MBP sampled both apo‐state and holo‐state, and RDCs were pruned to those of the above‐identified invariant residues. For our tests, we assumed that the two states being sampled were the apo‐state (1OMP) and the maltotriose‐bound holo‐state (3MBP). Synthetic RDCs for the invariant residues were generated using the alignment tensors obtained from best‐fitting the apo‐state RDCs to apo*, and the holo‐state RDCs to holo*.

**FIGURE 3:**
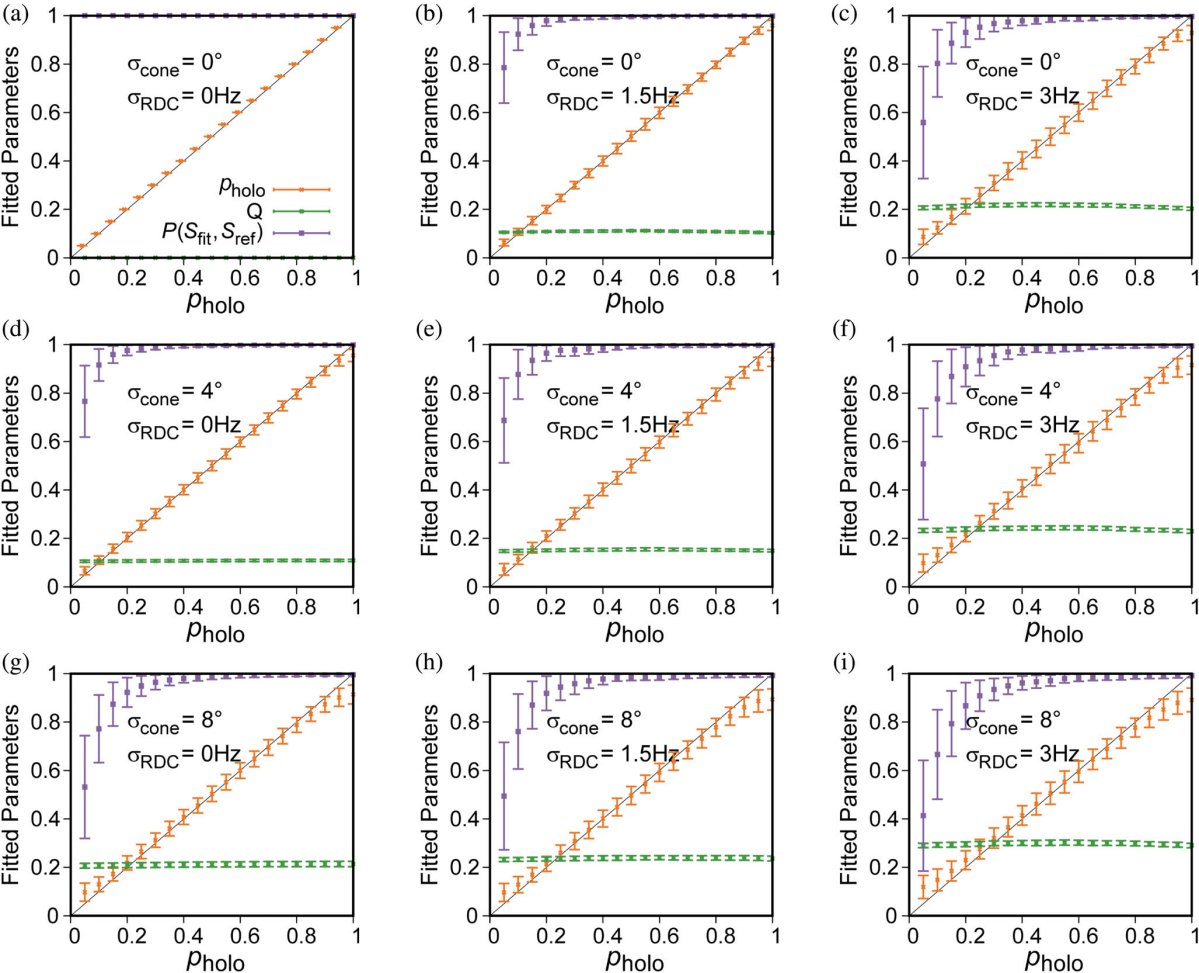
Simulated dependence of parameters obtained from a two‐state SVD fit on noise in RDCs (*σ*_RDC_) and structural coordinates (*σ*_cone_) for a dynamic two‐state MBP ensemble of apo‐ and holo‐states, with *p*_holo_ being the population of the holo‐state (see section 4.4). Parameters obtained from the two‐tensor fit are plotted against the input population of the holo‐state (*p*_holo_): the fitted population of the holo‐state (orange), the normalized scalar product P(***S***_fit_, ***S***_ref_) between the fitted and input tensors for the holo‐state used to generate the simulated RDCs (purple), and the *Q* value (green). Note that the uncertainty in the fitted parameters increases strongly when the difference in domain orientation between apo‐ and holo‐states is reduced (Figure S3).

As expected, in the absence of added noise to the RDCs (*σ*_RDC_) or uncertainties in the atomic coordinates defining bond vector orientations (*σ*_cone_), the two‐state SVD perfectly quantified the relative populations, 1 − *p*_holo_ and *p*_holo_, when fitting a linear sum of (1 − *p*_holo_)RDC^apo^ + *p*_holo_ × RDC^holo^ to this set of two structures in the absence of noise (Figure 3a). Addition of Gaussian‐distributed noise to the RDCs introduced uncertainty in the derived fraction of holo‐state (orange symbols in Figure 3), while also showing a small but systematic overestimate of the minor population, and increased *Q* values (green symbols; Figure 3b,c). Introducing structural noise by adding 4° or 8° random error to the orientation of the bond vectors increases the uncertainty in extracted populations and *Q* values in a similar way (Figure 3d–i). The small but systematic overestimate of the minor state population was correlated with the deviation of the best‐fitted alignment tensor for this minor state from the input value used to generate the simulated data, as reflected in P(***S***_fit_, ***S***_ref_) < 1 (purple symbols in Figure 3). As can be seen from these simulations, the relative populations of apo‐state and holo‐state can be determined quite well when they differ by less than a factor of about five. However, to accurately extract lower populations of the minor state, both the experimental RDCs as well as the structural data need to be of very high quality, that is, *Q* < ~0.2.

We note that the uncertainty in the population of the minor state becomes more sensitive to errors in the RDCs and structural coordinates when the difference between the major and minor states gets smaller. For a minor state that is about halfway in between the apo‐state and holo‐state X‐ray structures, an analysis analogous to that of Figure 3 shows uncertainties in the population that are nearly four times larger and a larger systematic overestimate of the minor state population (Figure S3).

### 2.6 RDC grid search of two states

**FIGURE 4:**
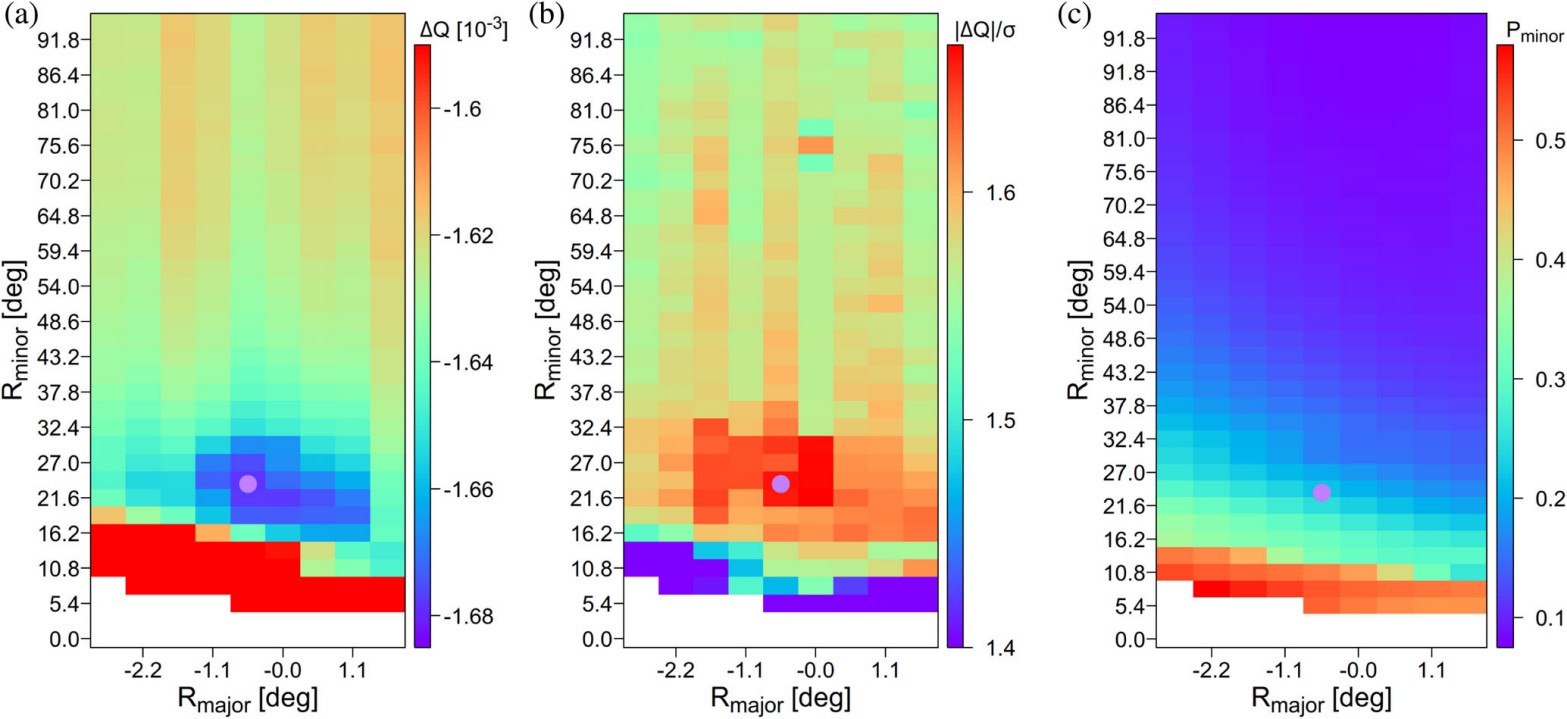
Results of a two‐tensor SVD grid search of apo‐RDCs (^1^D_CaC′_, ^1^D_C′N_, and ^1^D_NH_) of the selected invariant residues to two‐conformer MBP ensembles. Using the apo** version of the X‐ray structure (PDB id: 1OMP) as reference, the major conformer is generated by rotating the C‐terminal domain by a small angle, *R*_major_, about the (*θ*_*ρ*_, *ψ*_*ρ*_) rotation axis (see section 2.3); the second conformer rotated the C‐terminal domain by *R*_minor_ about this same axis. (a) Color map of the decrease in *Q* relative to a single‐conformer fit of apo**. The lowest grid point is marked by a pink circle. (b) The drop in Δ*Q*, normalized by its uncertainty *σ*(Δ*Q*). (c) Population of the minor conformer. Blank regions in the plots correspond to pairs of conformations that are too close to one another for unique identification by a two‐conformer SVD fit.

While the above analysis pointed to a slightly better single‐tensor RDC fit when the C‐terminal domain of the apo‐state was rotated by ~2.7°, it seemed likely that this rearrangement relative to the X‐ray crystal structure reflected the transient population of (partially) closed states, identified previously by PRE (Tang et al. 2007). For evaluating both the populations and domain orientations of such an equilibrium, we carried out a two‐state SVD fit of a 2D grid of conformers where the C‐terminal domain orientations of the apo* structure were rotated by *R*_major_ and by *R*_minor_ degrees about the rotation axis used for co‐aligning the tensors of the N‐ and C‐domains. The *R*_major_ grid search extended over a small range from −3° to +2°, to keep the major conformer close to the dominant apo‐state, whereas *R*_minor_ rotations extended up to ~90°, well beyond the angle where steric clashing between the domains would occur. For each such (*R*_major_, *R*_minor_) grid point, a two‐tensor SVD fit of RDCs of the invariant residues was carried out to find the populations of minor and major conformers that yielded an optimal two‐tensor SVD fit of the apo‐RDCs (Figure 4a).

**FIGURE 5:**
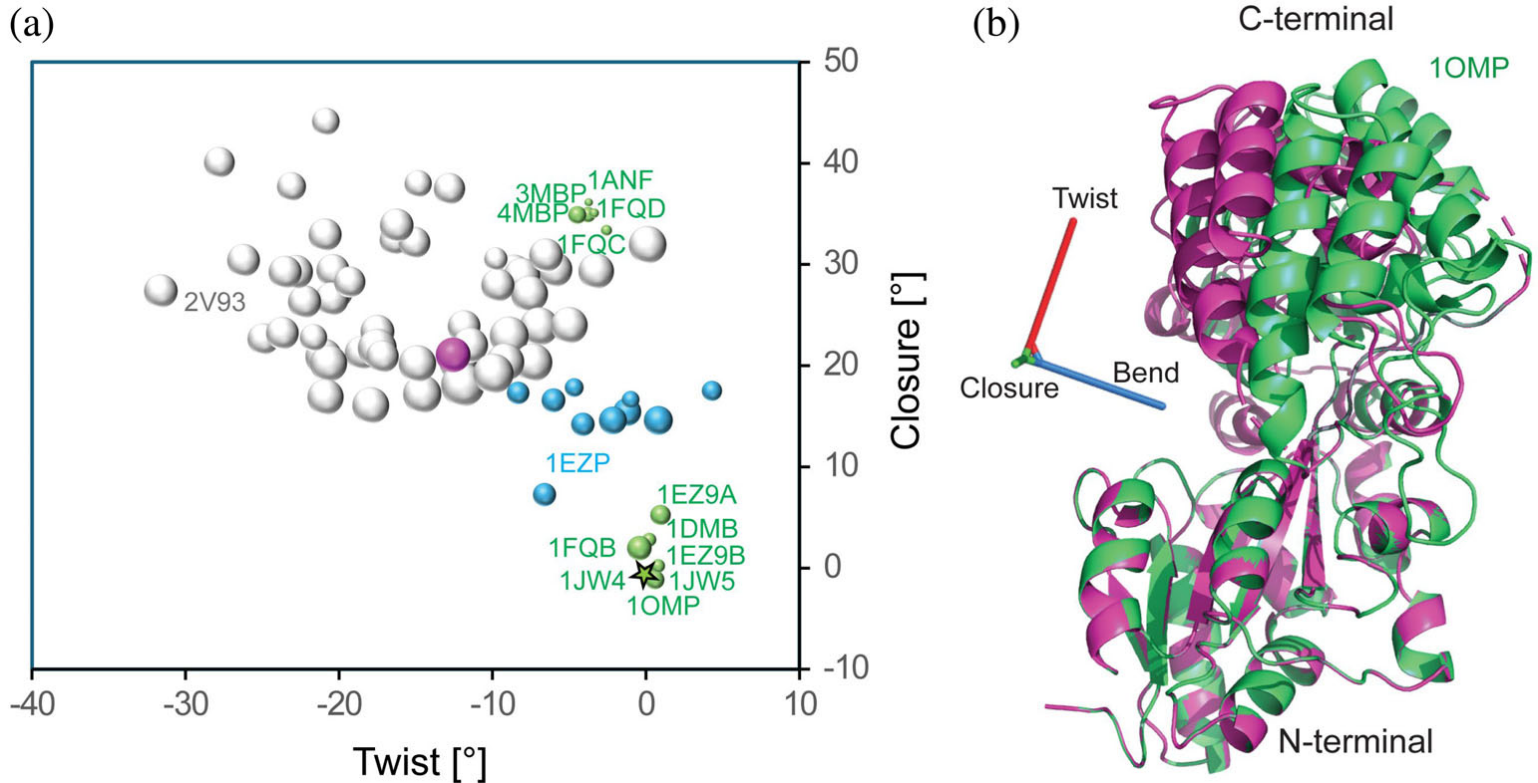
Relative orientation of MBP C‐terminal and N‐terminal domains. (a) Distribution of the relative orientations of the C‐terminal and N‐terminal domains in different states of MBP. For a set of MBP structures, including X‐ray structures (green spheres) with an open conformation (1DMB, 1JW4, 1JW5, 1FQB, and 1EZ9), X‐ray structures with a closed conformation (3MBP, 4MBP, 1ANF, 1FQC, and 1FQD), 50 conformers of an ensemble refined with PRE data (2V93) (gray; Tang et al. 2007), 10‐conformers of an RDC‐refined ensemble of β‐cyclodextrin bound MBP (1EZP; Mueller et al. 2000) (blue), and our RDC‐optimized minor apo‐MBP state (purple). Plots show combinations of closure and twist angles of the C‐terminal domain, with the area of the spheres proportional to the bend angle (12° for the purple sphere). The reference apo‐MBP (1OMP) is shown as the green star; see Figure 1 for definitions of the closure, twist, and bending axes. (b) Ribbon diagrams of apo‐MBP (1OMP) (green) and the minor conformation (purple) obtained from the grid search.

With *Q* = 0.1674, the grid search yielded improved agreement with the RDCs over the fits to the static, intermediate apo** model, albeit by an amount that was only 1.65 times larger than the statistical uncertainty in *Q* (Figure 4b). At the lowest grid point of the search, the minor conformation yielded a population of 16 ± 7% and a rotation angle *R*_minor_ of 25 ± 6° for its C‐terminal domain (Figures 4 and 5). In the nomenclature of the previously defined closure, twist, and bend axes (Evenäs et al. 2001b), these correspond to angles of 21°, −13°, and 12°, respectively (Figure 5a). The decrease of 0.0017 in *Q* for the two‐tensor fit over a single‐tensor fit to apo** (*Q* = 0.1691) is 1.65 times larger than the statistical uncertainty (~0.0010) in *Q* obtained from jackknife analysis. Using a standard statistical *F* test, this improvement corresponds to *F* = 0.08, or significance at the 8% level. Similarly, Akaike information criterion (AIC) analysis (Akaike 1974) comparing the single‐ and two‐tensor fits yielded a small but positive AIC value of 0.04, indicating that the two‐tensor model indeed is better than the standard single‐tensor model SVD fit, albeit by a small margin.

Remarkably, without restricting the fit to invariant residues, a very similar C‐terminal domain reorientation is found to yield the best fit to the RDCs, but the improvement in fit quality then is only 0.65 times the statistical uncertainty of 0.0016 in *Q* (Figure S4e) and no longer statistically significant (AIC = −5.4). This result indicates the importance of restricting the fits to peptide planes whose coordinates are invariant within the selected domains.

An alternate method for evaluating whether the two‐tensor fit represents a better model than a single tensor utilizes jackknifing, where indeed the two‐tensor model provides an improved prediction of RDCs not used in the fit (Figure S5), albeit by a very small margin. When carrying out a jackknifed two‐tensor fit for the holo* structure, an increase in *Q*_*jk*_ is obtained over that of a single‐tensor fit (Figure S6), which indicates that for the holo‐state the two‐state model is not warranted by the data.

Both grid searches also revealed that for grid points where the major and minor conformers were insufficiently different from one another (blank spaces in Figure 4), jackknifed two‐tensor SVD fits yielded widely diverging results with very large and nearly linearly opposing alignment tensors (Figure S5). These aberrant fits are a consequence of the fact that for small domain excursions, the time‐averaged RDCs for a two‐state ensemble become indistinguishable from those predicted for a static time‐averaged interdomain orientation.

To prevent inclusion of such degenerate fit results, we enforced a threshold on the sum of the magnitudes of the two fitted tensors, such that the Σ(|*D*_*a*_|) remains smaller than 2|*D*_*a*,ref_|, where *D*_*a*,ref_ is the alignment strength of the corresponding single‐tensor SVD fit of the RDC data.

## 3 DISCUSSION

A common concern in the use of liquid crystals to study a flexible system is that the steric and electrostatic forces that underlie the alignment could distort the conformational equilibrium. However, we note that the energies needed to generate the extremely weak degree of alignment needed to yield detectable RDCs, corresponding to alignments that are scaled down by more than a thousand‐fold relative to the static molecule, are about three orders of magnitude smaller than *k*T and therefore unlikely to impact conformational equilibria to a measurable extent. For example, a study on the relative domain orientation of a flexibly hinged T4 lysozyme mutant found the same time‐averaged conformation in steric and electrostatic alignment media, which fell roughly halfway in between two clusters of closed and open state X‐ray structures available for this protein (Goto et al. 2001).

Our analysis of high‐quality MBP RDCs, previously recorded by members of the Kay laboratory, was used to evaluate whether two‐tensor fitting can be used to identify structural equilibria. For the apo‐state of the protein, RDCs showed improved agreement for a two‐state structural equilibrium over any single, static structure. Analogous analysis of the MBP holo‐state RDCs indicated that these data were in excellent agreement with a single, closed state that was indistinguishable from its X‐ray structure (3MBP). Therefore, the population of any transient open MBP state, required to allow substrate release, was too small to be detected by a two‐state RDC analysis.

Two‐state analysis of the RDCs required high quality of the experimental RDC values plus the use of highly stringent criteria to identify invariant domains, with the orientations of internuclear vectors averaged over multiple high‐resolution X‐ray structures to reduce the effect of structural noise. Analysis of the large number of high‐quality RDCs available for the apo‐state of MBP yielded a best‐fit for an ensemble where the major conformer was very close to that of the apo‐state X‐ray structure (1OMP) and a minor state that was tilted towards that of the maltotriose‐bound state (3MBP) but did not reach this closed holo‐state (Figure 5). The relative domain orientation of this minor state falls near the center of the ensemble of NMR structures previously derived from a combination of RDC and PRE data (gray spheres in Figure 5a). Notably, PREs derived for our two‐state ensemble are remarkably similar to those reported for the previously reported ensemble of conformers that was refined on the basis of PRE data (Figure S7) (Tang et al. 2007). Our result therefore suggests that analysis of such two‐state equilibria can be feasible without introducing paramagnetic tags. However, prerequisites for such analysis are (a) the availability of large numbers of accurately measured backbone RDCs for each domain, (b) the ability to identify “invariant” residues within each domain, with their vector orientations averaged over multiple X‐ray structures to increase the precision of their intra‐domain orientation, (c) a substantial population (>~10%) of the minor state, and (d) a substantial difference in interdomain orientation (>~20°). Even while for the holo‐state of MBP there must exist “open” states where ligand can be released, our analysis of holo‐state RDCs found no improvement in their fit when searching for such a two‐state equilibrium, indicating that the population of the open state must fall below the limits detectable by RDC analysis. Although this result is consistent with prior conclusions based on PRE analysis, the latter was reported to be rather insensitive to minor apo‐state populations for the ligated protein because an open state did not introduce unique short PRE distances (Tang et al. 2007).

Our analysis of RDCs acquired in a single‐alignment medium demonstrates that it can reveal domain motion even when the amplitude of such motion is fairly small and its population is low. For larger amplitude motions, tagging one domain with a paramagnetic lanthanide and comparing the induced magnetic alignments of the protein's different domains presents an elegant and powerful method to identify domain motion (Barnes et al. 2019; Bertini et al. 2004; Eichmueller and Skrynnikov 2007; Karschin et al. 2022). However, for a case such as MBP, where the amplitude of the C‐domain motion relative to the N‐domain is modest and the population of the minor state is low, the *D*_*a*_ value of the C‐domain relative to a paramagnetically aligned N‐domain is only reduced by ~1.4%, which can be difficult to establish at sufficient precision. In particular, we note that high precision of the RDCs can be a concern in paramagnetically aligned proteins whose spectra are adversely impacted by paramagnetic line broadening. On the other hand, the sensitivity to domain motions can be enhanced by measurement of relaxation dispersion of the paramagnetic shifts, provided the motions are sufficiently slow to be revealed by such experiments (Stiller et al. 2022).

While we represent the conformation of apo‐MBP as a dynamic equilibrium between two static conformers, we point out that this is the minimum number of conformers needed to optimally fit the RDC data. Clearly, both conformers are dynamic entities with fluctuations of both atomic positions and domain orientations around their time‐averaged values. However, these fluctuations do not give rise to substantial non‐uniform scaling of the RDCs and therefore cannot be uniquely extracted from such data.

Domain dynamics of apo‐MBP also has been investigated by both accelerated molecular dynamics (Bucher et al. 2011) and by coarse‐grained molecular dynamics simulations (Wang et al. 2012). Although time scales in such studies tend to be strongly shortened, the authors extrapolate that transitions take place on a sub‐microsecond time scale, consistent with the absence of relaxation dispersion which was interpreted as consistent with a time scale of 20 ns to 20 μs for the domain rearrangement (Tang et al. 2007). No information on the time scale of domain dynamics can be gleaned from the RDC data, other than that the dynamics must be sufficiently fast (<~500 μs) to prevent extensive broadening of NMR resonances.

Two‐state equilibria such as present for apo‐MBP are common in regulated enzymes as well as signal‐transduction proteins. It therefore appears likely that two‐state equilibria can be accurately probed by advanced RDC measurements, provided that high‐resolution crystal structures are available for potential “end states,” and that the smallest invariant unit is large enough for the measurement of >~100 high‐quality RDCs. The latter often requires the use of perdeuterated protein and inclusion of multiple RDCs other than the commonly measured ^15^N‐^1^H backbone amide couplings.

## 4 METHODS

### 4.1 Quality factor, *Q*

A quality factor, *Q*, is commonly used to evaluate the quality of the fits between RDCs and molecular coordinates,

(5) $$Q=\sqrt{\sum_{i=1\ldots N}\frac{{\left(D_{i}^{\text{pred}}-D_{i}^{\text{meas}}\right)}^{2}}{\left[N\left\{\frac{D_{a}^{2}\left(4+3{\mathrm{Rh}}^{2}\right)}{5}\right\}\right]},}$$

where *i* extends over all *N* normalized RDCs used in the fit. Since an alignment tensor has five adjustable parameters, doubling to 10 when carrying out two‐tensor fits, jackknifing can be used to prevent overfitting (Clore and Garrett 1999). For this purpose, for *N* RDCs, the *Q* factor calculation is repeated *N* times, each time omitting a different RDC that is then used for deriving $D_{i}^{\text{meas}}-D_{i}^{\text{pred}}$, the difference between measured and predicted values. The jackknifed quality factor is then calculated from

(6) $$Q_{\mathrm{jk}}=\sqrt{\sum_{i=1\ldots N}\frac{{\left(D_{i}^{\text{pred}}-D_{i}^{\text{meas}}\right)}^{2}}{N\left\{D_{a,i}^{2}\left(4+3{\mathrm{Rh}}_{i}^{2}\right)/5\right\}},}$$

where *D*_*a*,*i*_ and *Rh*_*i*_ are the alignment strength and rhombicity obtained when the *i*th RDC is omitted from the SVD fit. When fitting a large number (N≫5) of RDCs, *Q*_*jk*_ approaches the standard *Q* value, obtained when including all RDCs in a single SVD fit (Shen et al. 2023).

For a two‐tensor fit, the use of the generalized alignment tensor magnitude as the denominator in Equation (6) is no longer appropriate because the two tensors can have opposite signs, which can lead to very large *D*_*a*_ values even when RDCs are small. The use of such large *D*_*a*_ values in the two‐tensor analog of Equation (5) then can result in artificially small *Q* values. Therefore, the denominator is replaced by *N ×* RMS($D_{i}^{\text{meas}}$), used in earlier work, which approaches the denominator of Equation (5) for a one‐tensor fit when the RDC vector orientations are roughly uniformly distributed in orientational space (Bax 2003).

### 4.2 Peptide plane selections for RDC fits

Peptide planes included for RDC analysis were restricted to those that were most conserved within their respective N‐ and C‐terminal domains when comparing all nine high‐resolution X‐ray structures (1OMP, 1JW4, 1JW5, 1FQB, 1EZ9, and 1DMB for the apo‐state and 3MBP, 1ANF, and 4MBP for ligated states; see Table 1). In a first step, the MaxSub procedure (Siew et al. 2000) with a distance cutoff of 0.5 Å was used to identify the largest collection of residues in the N‐ and C‐terminal domains whose C^α^ coordinate RMSD from average in the aligned conformers was ≤0.5 Å.

In a second step, peptide planes within the best‐fit superimposed domains are identified whose C^α^‐C′, C′‐N, and N‐H vectors deviate by an RMS value ≤5° from their averaged orientation. This second round of selection resulted in a total of 198 peptide planes, 97 in the N‐domain and 101 in the C‐domain (Figure 1a). Cut‐off values for the coordinate and angular variation correspond to thresholds below which the number of invariant residues drops rather sharply (Figure S8).

### 4.3 Vector orientation averaging

To further reduce the impact of coordinate uncertainty on the two‐tensor fits, RDC bond vector orientations in the highest resolution apo‐state X‐ray structure (1OMP) were replaced by those of the averaged orientations of all 10 N‐terminal domain X‐ray structures. Specifically, (1) ten X‐ray structures, to which hydrogens were added by the Dynamo module of NMRPipe (Delaglio et al. 1995), were superimposed using backbone atoms of the selected invariant N‐terminal domain residues; (2) averaged unit vectors were then calculated for three types of bonds, N‐H, C′‐N, and C^α^‐C′, for each residue *i*, and denoted as <N(*i*)‐H(*i*)>, <C′(*i* − 1)‐N(*i*)>, and <C^α^(*i*)‐C′(*i*)>, respectively; (3) starting from the 1OMP X‐ray structure, all C′ atomic coordinates were preserved, while the C^α^(*i* − 1) and N(*i*) atoms were re‐positioned by using the averaged bond vector orientations of <C^α^(*i* − 1)‐C′(*i* − 1)> and <C′(*i* − 1)‐N(*i*)>, respectively, relative to the position of the fixed C′(*i* − 1) atom, while keeping bond lengths fixed at their 1OMP distance; (4) the H^N^(*i*) atom was added by using the <N(*i*)‐H(*i*)> vector relative to the re‐positioned N(*i*) atom.

The same substitution of bond vector orientations was used for the C‐terminal domain of 1OMP, and the apo‐structure of 1OMP with these substituted averaged vector orientations was used to generate apo*. Similar substitution of bond vectors in the holo‐state structure (3MBP) was used for generating a holo* structure.

### 4.4 Testing of the effect of noise on two‐tensor SVD fit

Simulated ^1^ *D*_HN_, ^1^ *D*_C′N_, and ^1^ *D*_CαC′_ RDCs, *D*, were generated for a two‐state ensemble of apo‐state (1OMP) and holo‐state MBP (3MBP) conformers, with populations 1 − *p*_holo_ and *p*_holo_,

(7) $$D=\left(1-p_{\text{holo}}\right)\times D_{\mathrm{apo}}+p_{\text{holo}}\times D_{\text{holo}}+D_{\text{noise}}\left(\sigma_{\mathrm{RDC}}\right).$$

Here, *D*_apo_ and *D*_holo_ are the synthetic ^1^ *D*_HN_, ^1^ *D*_C′N_, and ^1^ *D*_CαC′_ RDC values generated for the above‐identified invariant residues in apo* and holo*, but restricted to those that also had experimental RDCs in both apo‐ and holo‐states. Saupe alignment tensors ([*S*_*zz*_, *S*_*xx*_–*S*_*yy*_, *S*_*xy*_, *S*_*xz*_, *S*_*yz*_]) used for generating the RDCs were ***S***_apo_ = [9.7866, −3.1494, −3.4426, −4.9529, 9.1678] × 10^−4^ and ***S***_holo_ = [12.311, −9.2219, −3.6206, −1.4577, 5.8561] × 10^−4^, respectively, and correspond to the best‐fitted tensors obtained by fitting the apo‐RDCs to the invariant residues in apo*, and by fitting the holo‐RDCs to the selected invariant residues in holo* for holo MBP. *D*_noise_(*σ*_RDC_) represents Gaussian‐distributed noise with an RMS value *σ*_RDC_ Hz, added to the normalized synthetic RDCs.

Analogously, vector orientations were perturbed by a cone‐shaped distribution, with a standard deviation *σ*_cone_, and a relative probability of $\sin\left(\beta \right)\exp\left(-\beta^{2}/\sigma_{\text{cone}}^{2}\right)$ for an angle *β* between the original and modified orientation. For testing purposes, such a random change in vector orientations with amplitude *σ*_cone_ was added to all C^α^‐C′, C′‐N and N‐H vectors of the apo* and holo* conformers in the MBP ensemble.

### 4.5 Generation of representative ensemble

An ensemble representation containing the optimized states and populations obtained from the RDC grid search procedure (see section 2.6) was generated rather than the apo* coordinates which contain non‐ideal geometry resulting from the averaging of bond vectors. For each of 10 selected X‐ray structures (Table 1), a pair of open/closed structures was first generated, with the N‐terminal and C‐terminal domains each rotated to superimpose the corresponding domain in the optimal major(open)/minor(closed) conformation obtained from the grid search (Figures 4 and 5). The linker residues were then added to each structure of the above‐generated open/closed ensemble by using the IVM module in Xplor‐NIH (Schwieters and Clore 2001), with the coordinates of the N‐terminal and C‐terminal domains frozen, a van der Waals repulsion term to prevent atomic overlap, a hydrogen bond database potential (Schwieters et al. 2020), and weak harmonic *ϕ*/*ψ*/*ω* torsion angle restraints (10 kcal/rad^2^) within the linkers. The final version of the representative ensemble (PDB: 9PGN) contains 10 pairs of major(open)/minor(closed) conformers, each of which contains C‐terminal domains and linkers modified by XPLOR‐NIH, populated at 84% and 16%, representing the open and partially closed states, respectively. The N‐terminal domains for each pair of structures are identical to their corresponding X‐ray structure.

## AUTHOR CONTRIBUTIONS

**Yang Shen:** Investigation; software; methodology; writing – original draft; data curation; formal analysis. **Ad Bax:** Conceptualization; funding acquisition; writing – review and editing; supervision.

## CONFLICT OF INTEREST STATEMENT

The authors declare no conflicts of interest.

## Supporting information


**Data S1.** Supporting Information figures showing additional SVD and grid search results as well as predicted PRE profiles.

## Data Availability

The data that support the findings of this study are openly available in github at https://github.com/bax-group/DC-SVDM.
