# Supplementary material for: Two‐conformer equilibrium of maltose‐binding protein in the absence of ligand from residual dipolar coupling analysis
Source: Protein Sci. 2025 Dec 23;35(1):e70425. doi: 10.1002/pro.70425 (PMC12723730; doi:10.1002/pro.70425)
Supplement: Supplementary file 1 — Data S1. Supporting Information figures showing additional SVD and grid search results as well as predicted PRE profiles. [file PRO-35-e70425-s001.pdf]

Two-conformer equilibrium of maltose-binding protein in the absence of ligand from residual dipolar coupling analysis

Yang Shen and Ad Bax

Laboratory of Chemical Physics, National Institute of Diabetes and Digestive and Kidney Diseases, National Institutes of Health, Bethesda, MD 20892-0520, U.S.A

SUPPORTING INFORMATION

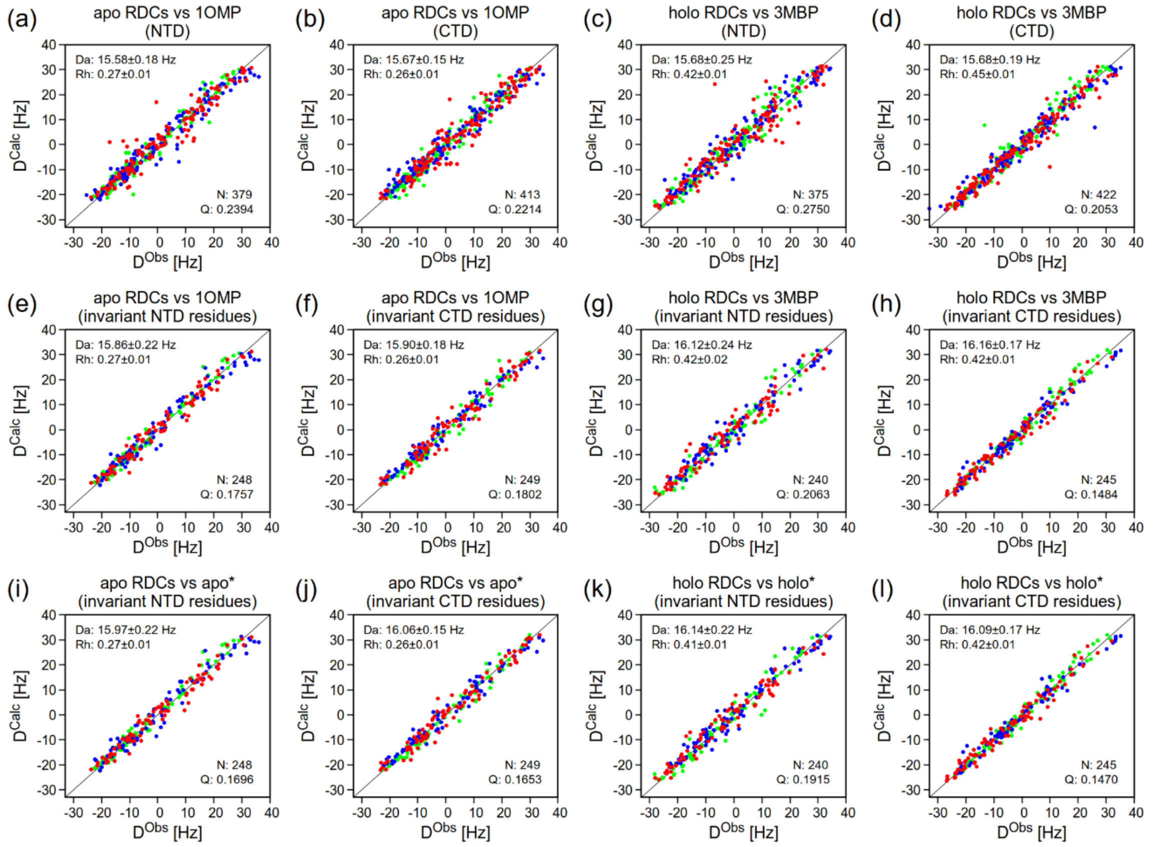

FIGURE S1. Best-fitted versus observed RDCs, with the fits carried out for individual MBP domains. The single-tensor SVD fits were performed separately for the N-terminal (1st and 3rd columns) and C-terminal (2nd and 4th columns) domains of (**a,b,e,f,i,j**) apo RDCs (from ref. (Yang et al. 1999)) vs apo-X-ray structure (1OMP) (Sharff et al. 1992), and (**c,d,g,h,k,l**) holo RDCs (from ref. (Evenäs et al. 2001)) vs holo Xray structure (3MBP) (Quiocho et al. 1997). $^1D_{NH}$ are shown in red, $^1D_{NC'}$ in green, and $^1D_{C'C\alpha}$ in blue, with $^1D_{NC'}$ and $^1D_{C'C\alpha}$ upscaled by factors of 5 and 8, respectively, to account for their intrinsic dipolar interaction strength. (**a-d**) Fits performed for all residues in N-terminal or C-terminal domains of MBP. (**e-h**) Fits performed for the pruned set of vector orientations (<0.5 Å; <5°; see Fig. 1A). (**i-l**) Fits performed for the selected pruned vectors after they were replaced by orientations averaged over ten high-resolution X-ray structures (see Methods).

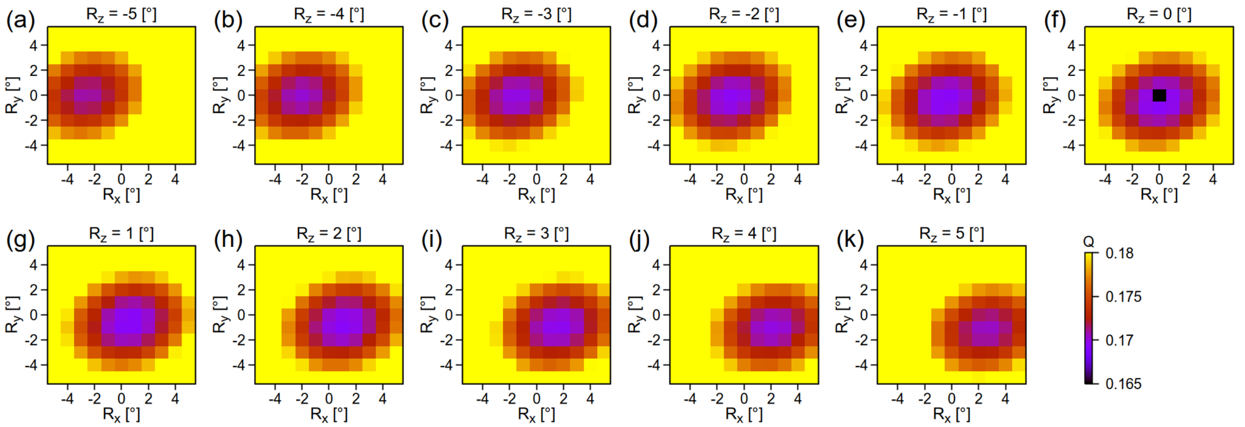

FIGURE S2. Results of a single-tensor SVD fit apo-RDCs ($^{1}D_{C\alpha C'}$, $^{1}D_{C'N}$ and $^{1}D_{NH}$) to apo-MBP with different domain orientation. The single-tensor SVD fits were performed for the pruned set of vector orientations (<0.5 Å; <5°; see Fig. 1a) and the apo** conformer (see Results 2.3) after rotating the C-terminal domain by small angles about x-, y- and z, referred as R_x, R_y and R_z, respectively. (**a-k**) Color map of the fitted Q factors plotted for C-terminal domain rotations by R_y vs R_x, for each R_z angle. A lowest Q factor of 0.1691 is observed in panel f for the reference apo** conformer (R_x, R_y, R_z = 0). The corresponding R_x, R_y and R_z rotations for generating the apo* conformer are 0.1°, 2.5°, 1.9°, respectively.

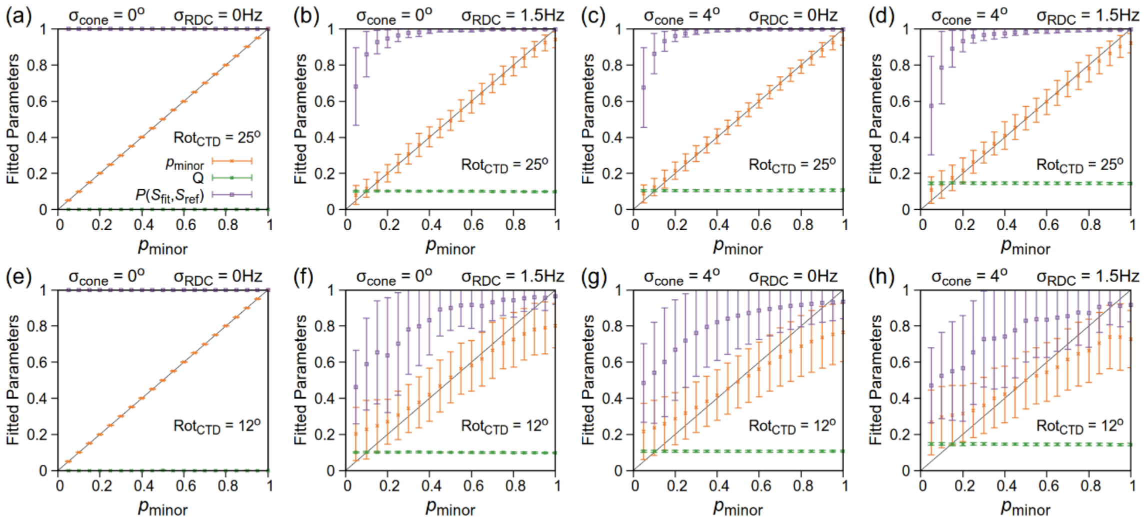

FIGURE S3. Simulated dependence of parameters obtained from a two-state SVD fit on noise in RDCs (σ_{RDC}) and structural coordinates (σ_{cone}) for a dynamic two-state MBP ensemble of apo* and a second structure, referred as the minor structure, where the C-terminal domain is rotated by (**a-d**) 25° and (**e-h**) 12° about the (ϕ_r,ψ_r) axis (Section 2.2), with p_{minor} being the population of the minor state (see Section 4.4). Fits are shown (**a,e**) in the absence of RDC and structural noise; (**b,f**) in the presence of 1.5 Hz RDC uncertainty; (**c,g**) in the presence of 4° structural noise; and (**d,h**) in the presence of both 1.5 Hz RDC uncertainty and 4° structural noise. Parameters obtained from the two-tensor fit are plotted against the input population of the minor-state (p_{minor}): the fitted population of the minor-state (orange), the normalized scalar product P($\boldsymbol{S}_{fit}$, $\boldsymbol{S}_{ref}$) between the fitted and input tensors for the minor state used to generate the simulated RDCs (purple), and the Q value (green).

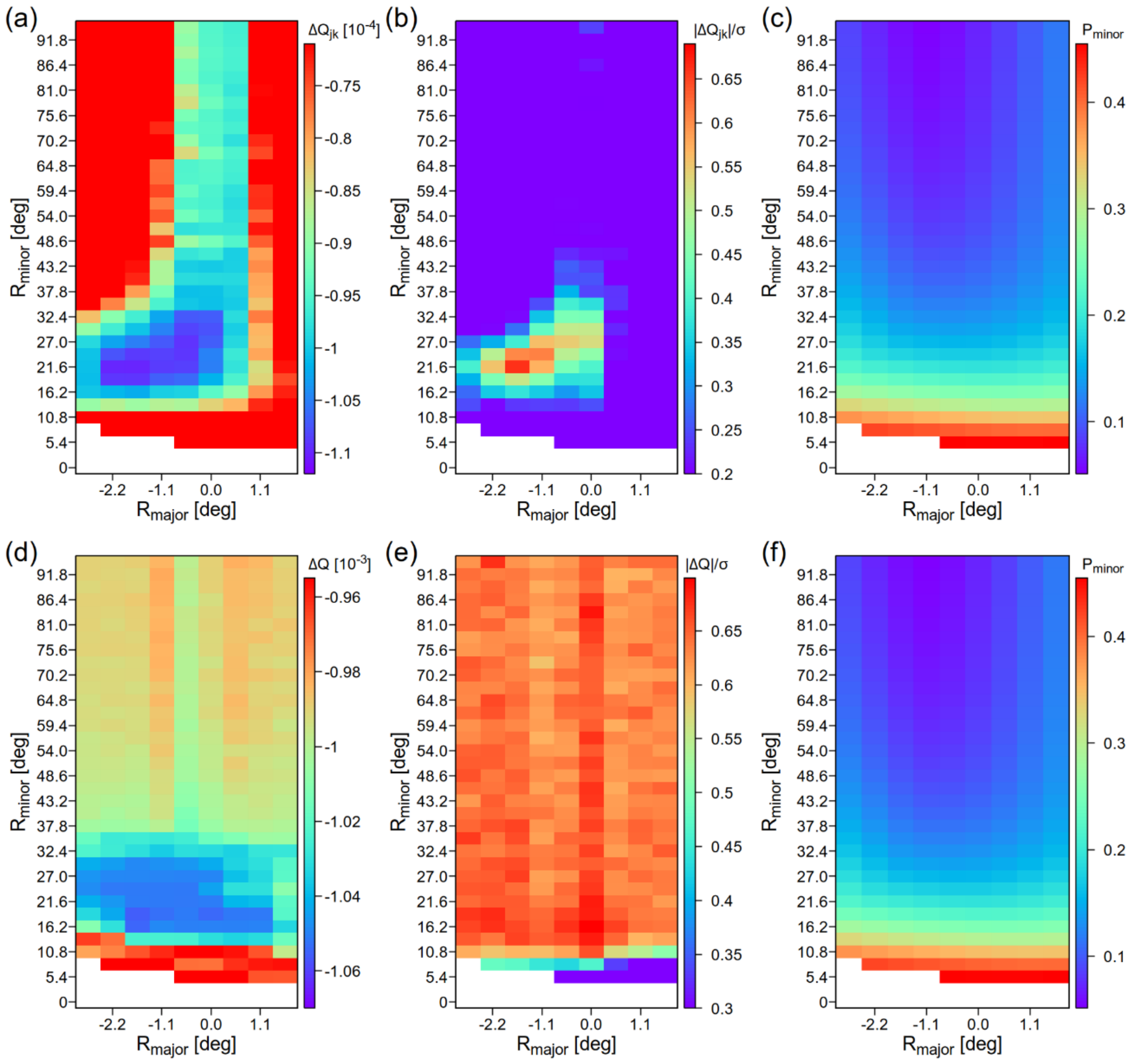

FIGURE S4. Results of a two-tensor SVD grid search to two-conformer MBP ensembles of all 792 apo-RDCs ($^1D_{C\alpha C'}$, $^1D_{C'N}$ and $^1D_{NH}$). Using the apo** version of the X-ray structure (PDB id: 1OMP) as reference, the major conformer is generated by rotating the C-terminal domain by a small angle, R_{major}, about the (θ_r,ψ_r) rotation axis (see section 2.3); the second conformer rotated the C-terminal domain by R_{minor} about this same axis. (**a**) Color map of the decrease in Q_{jk} relative to a single-conformer fit of apo**. The lowest grid point is marked by a pink circle. (**b**) The drop in ΔQ_{jk}, normalized by its uncertainty $\sigma Q_{jk} = \sigma(\Delta Q_{jk}) \times \sqrt{N_{jk}}$ of all N_{jk} jack-knifed fittings, where $\sigma(\Delta Q_{jk})$ represents the standard deviation in ΔQ_{jk} among the jackknifed fits. (**c**) Population of the minor conformer. Blank regions in the plots correspond to pairs of conformations that are too close to one another for unique identification by a two-conformer SVD fit. (**d-f**) Analogous to (a-c) but with the non-jackknifed Q value used for the plots.

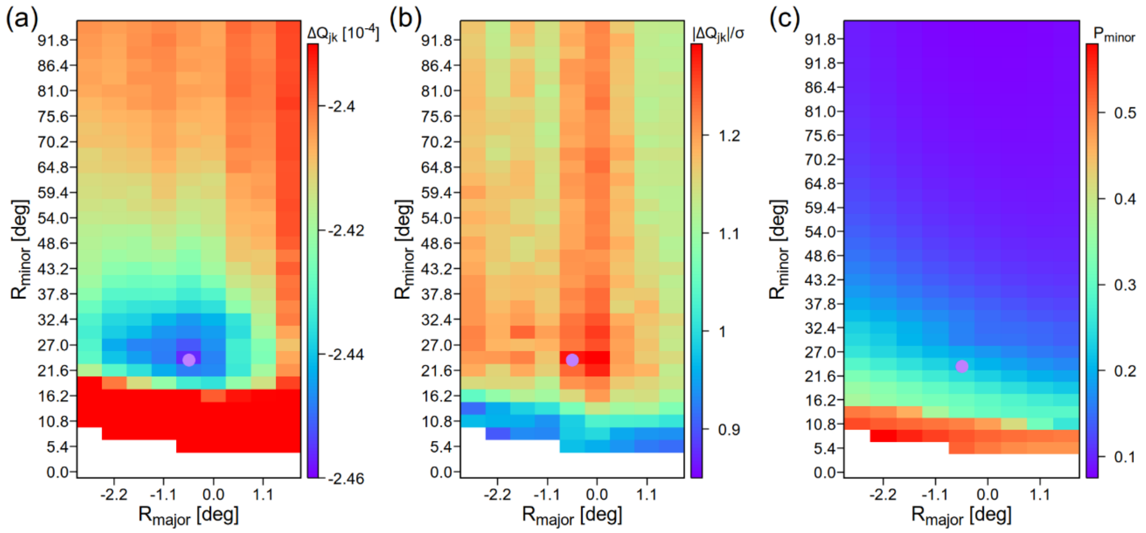

FIGURE S5. Results of a two-tensor SVD grid search of apo-RDCs ($^1D_{C\alpha C'}$, $^1D_{C'N}$ and $^1D_{NH}$) of the invariant residues to two-conformer MBP ensembles using jackknifed Q_{jk} values. Using the apo* version of the X-ray structure (PDB id: 1OMP) as reference, the major conformer is generated by rotating the C-terminal domain by a small angle, R_{major}, about the (θ_r,ψ_r) rotation axis (see Section 2.3); the second conformer rotated the C-terminal domain by R_{minor} about this same axis. (**a**) Color map of the decrease in Q_{jk} relative to a single-conformer fit of apo*. The lowest grid point is marked by a pink circle. (**b**) The drop in ΔQ_{jk}, normalized by its uncertainty $\sigma Q_{jk} = \sigma(\Delta Q_{jk}) \times \sqrt{N_{jk}}$ of all N_{jk} jack-knifed fittings, where $\sigma(\Delta Q_{jk})$ represents the standard deviation in ΔQ_{jk} among the jackknifed fits. (**c**) Population of the minor conformer. Blank regions in the plots correspond to pairs of conformations that are too close to one another for unique identification by a two-conformer SVD fit.

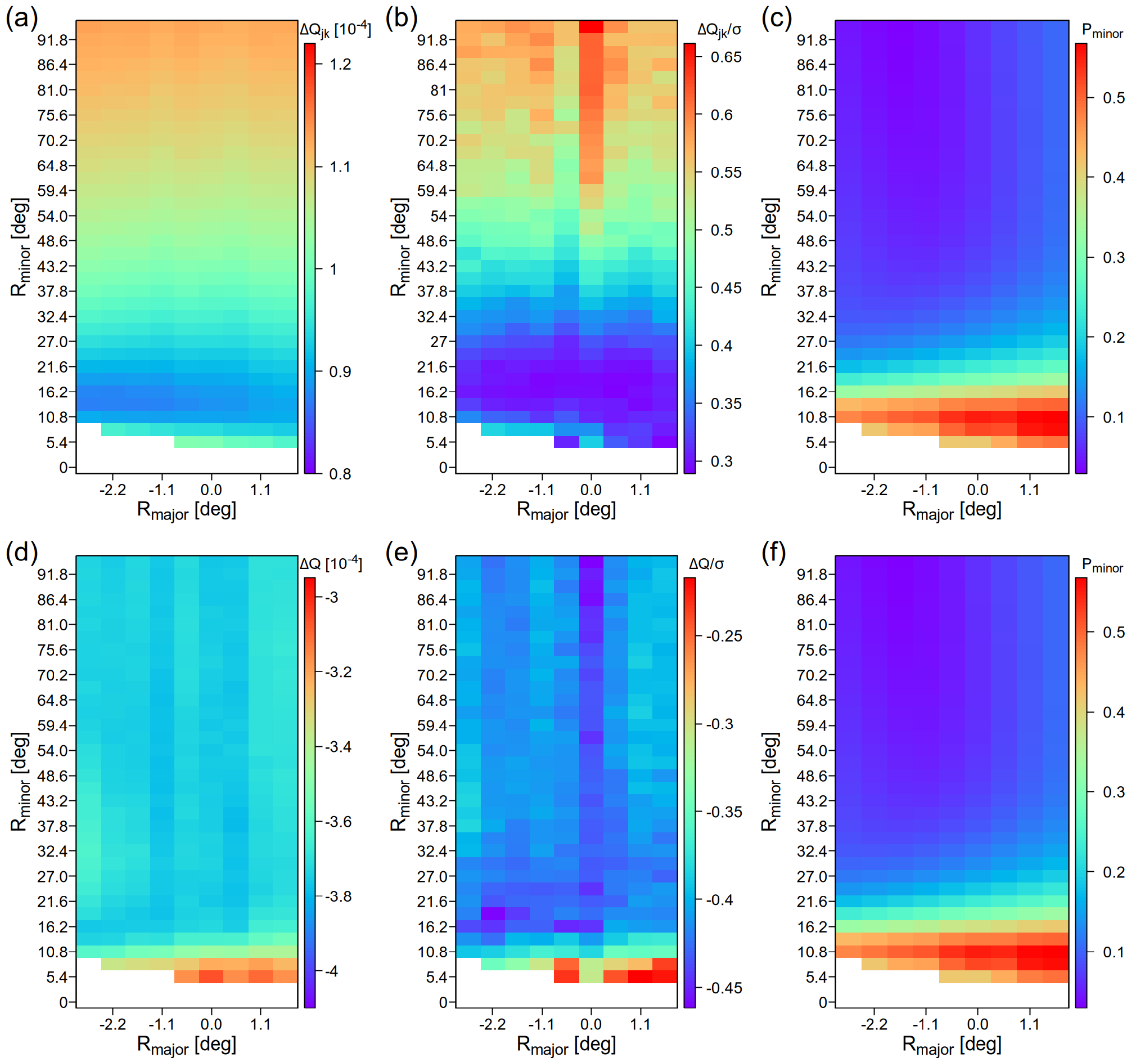

FIGURE S6. Results of a two-tensor SVD grid search of holo-RDCs ($^1D_{C\alpha C'}$, $^1D_{C'N}$ and $^1D_{NH}$) of the invariant residues to two-conformer MBP ensembles. Using the holo** version of the X-ray structure (PDB id: 3MBP) as reference, the major conformer is generated by rotating the C-terminal domain by a small angle, R_{major}, about the (θ_r,ψ_r) rotation axis (see section 2.3); the second conformer rotated the C-terminal domain by R_{minor} about this same axis. (**a**) Color map of the decrease in Q_{jk} relative to a single-conformer fit of holo**. (**b**) The drop in ΔQ_{jk}, normalized by its uncertainty $\sigma Q_{jk} = \sigma(\Delta Q_{jk}) \times \sqrt{N_{jk}}$ of all N_{jk} jack-knifed fittings, where $\sigma(\Delta Q_{jk})$ represents the standard deviation in ΔQ_{jk} among the jackknifed fits. (**c**) Population of the minor conformer. Blank regions in the plots correspond to pairs of conformations that are too close to one another for unique identification by a two-conformer SVD fit. (**d-f**) Analogous to (a-c) but with the non-jackknifed Q value used for the plots. The lowest Q and Q_{jk} values obtained from the two-tensor SVD fit are 0.1702 and 0.1725, respectively, while the Q and Q_{jk} values obtained from a single-tensor SVD fit of the holo** conformer are 0.1706 and 0.1724, respectively.

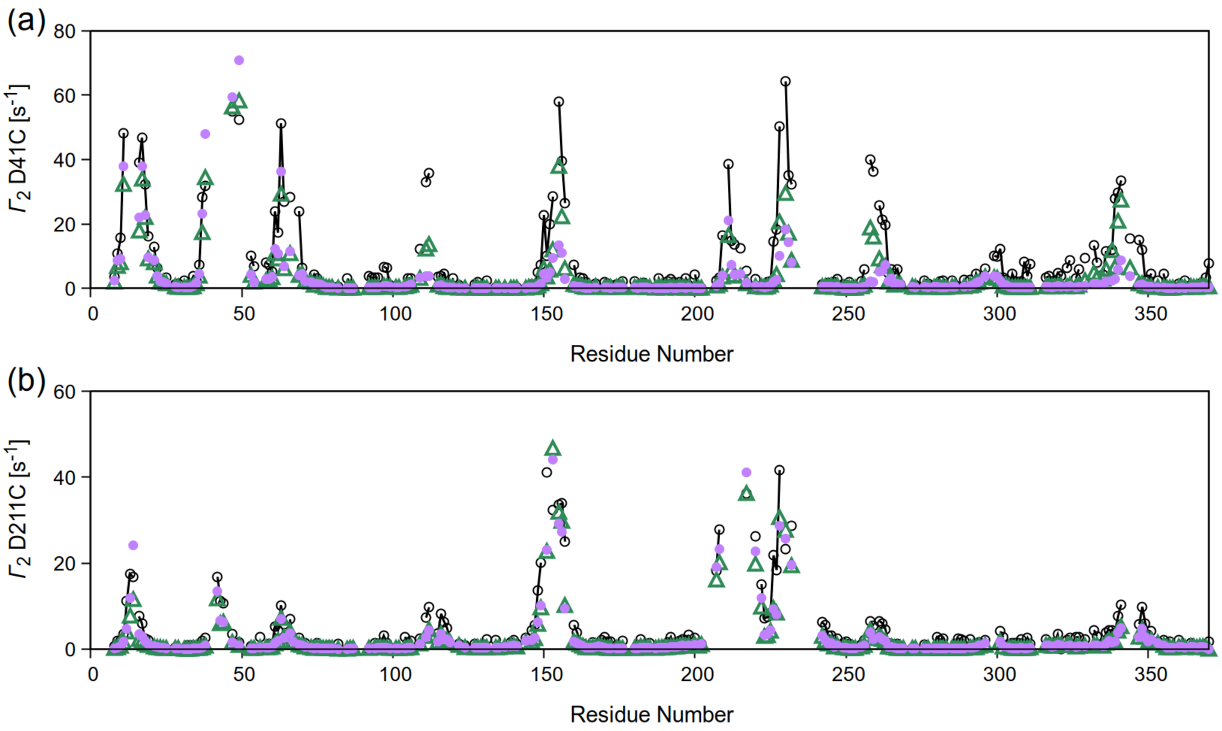

FIGURE S7, Comparison of observed (○ and lines), calculated PRE profiles for D41C (**a**) and S211C (**b**) for a 5% minor species population of 2V93 (Δ) and a 16% minor conformation population of the optimal ensemble in this work (●).

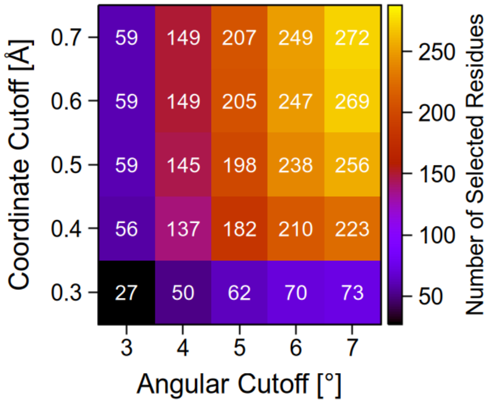

FIGURE S8. Number of the selected invariant residues in MBP X-ray structures obtained when different angular and coordinate variation cutoffs are used (see Section 4.2 for details).

References

Evenäs J, Tugarinov V, Skrynnikov NR, Goto NK, Muhandiram R, Kay LE Ligand-induced structural changes to maltodextrin-binding protein as studied by solution NMR spectroscopy. J Mol Biol 2001;309:961-974.

Quiocho FA, Spurlino JC, Rodseth LE Extensive features of tight oligosaccharide binding revealed in high-resolution structures of the maltodextrin transport chemosensory receptor. Structure 1997;5:997-1015.

Sharff AJ, Rodseth LE, Spurlino JC, Quiocho FA Crystallographic evidence of a large ligand-induced hinge-twist motion between the 2 domains of the maltodextrin binding protein involved in active transport and chemotaxsis. Biochemistry 1992;31:10657-10663.

Yang DW, Venters RA, Mueller GA, Choy WY, Kay LE TROSY-based HNCO pulse sequences for the measurement of (HN)-H-1-N-15, N-15-(CO)-C-13, (HN)-H-1-(CO)-C-13, (CO)-C-13-C-13(alpha) and (HN)-H-1-C-13(alpha) dipolar couplings in N-15, C-13, H-2-labeled proteins. J Biomol NMR 1999;14:333-343.
